# Supplementary material for: Lingering effects of COVID-19 on the mental health of first-year university students in Japan
Source: PLoS One. 2022 Jan 12;17(1):e0262550. doi: 10.1371/journal.pone.0262550 (PMC8754334; doi:10.1371/journal.pone.0262550)
Supplement: S1 Appendix — (PDF) [file pone.0262550.s001.pdf]

以下の項目は、誰もが持ちうる考えや気持ち、体験について書いています。それぞれの項目がどのくらい過去2週間のあなたに当てはまるか、「全く当てはまらない（0）」から「かなり当てはまる（4）」の中から選び、番号にマークしてください。
それぞれの文章をよく読み、1つの項目につき回答を1つだけ選び、すべての設問に答えてください。

		全く当てはまらない				かなり当てはまる
1	自分の家族のことを考えると、悲しくなったり怒りを感じたりする	0	1	2	3	4
2	人前では内気である	0	1	2	3	4
3	心配していることがたくさんある	0	1	2	3	4
4	はっきりとした理由もないのに胸がドキドキする	0	1	2	3	4
5	食べると自分をコントロールできないと感じる	0	1	2	3	4
6	授業を楽しめている	0	1	2	3	4
7	家族は自分のことを愛してくれていると思う	0	1	2	3	4
8	自分が自分でない感じがする	0	1	2	3	4
9	人と一緒にいても以前ほど楽しくない	0	1	2	3	4
10	ひとりぼっちで孤独だと感じる	0	1	2	3	4
11	家族のことでいら立つ	0	1	2	3	4
12	現実感がない	0	1	2	3	4
13	食べ物のことばかり考えてしまう	0	1	2	3	4
14	人前でパニック発作が起こるのではないか心配である	0	1	2	3	4
15	皆の前で話さなければならない時に不安になる	0	1	2	3	4
16	（中々眠れない、夜中に目が覚めるなど）睡眠に問題がある	0	1	2	3	4
17	色々な考えが次々と頭に浮かんでくる	0	1	2	3	4
18	自分の体型に満足している	0	1	2	3	4
19	自分には価値がないと感じる	0	1	2	3	4
20	自分の家族は幸せだと思う	0	1	2	3	4
21	自分の体重に満足していない	0	1	2	3	4
22	自分は無力だと感じる	0	1	2	3	4
23	食べすぎてしまう	0	1	2	3	4
24	頻繁に酒（アルコール）を飲む	0	1	2	3	4
25	恐怖やパニックに陥ることがある	0	1	2	3	4
26	酒（アルコール）を飲むと後で何が起こったか思い出せない	0	1	2	3	4
27	緊張している	0	1	2	3	4

以下の項目は、誰もが持ちうる考えや気持ち、体験について書いています。それぞれの項目がどのくらい過去2週間のあなたに当てはまるか、「全く当てはまらない（0）」から「かなり当てはまる（4）」の中から選び、番号にマークしてください。
それぞれの文章をよく読み、1つの項目につき回答を1つだけ選び、すべての設問に答えてください。

		全く当てはまらない				かなり当てはまる
28	食べはじめると止まらない	0	1	2	3	4
29	怒りを抑えるのが難しい	0	1	2	3	4
30	怯えたり驚いたりしやすい	0	1	2	3	4
31	頻繁にダイエットをしている	0	1	2	3	4
32	友だちを作るのが得意である	0	1	2	3	4
33	時々、何かを壊したい気持ちになる	0	1	2	3	4
34	好ましくないことを考えてしまい、コントロールできない	0	1	2	3	4
35	うちの家族には虐待があった	0	1	2	3	4
36	悪夢を見たり、フラッシュバックがある	0	1	2	3	4
37	いつも悲しい	0	1	2	3	4
38	他人から嫌われていないかと心配である	0	1	2	3	4
39	もっと自分の家族が仲良くしていたら良いのにと思う	0	1	2	3	4
40	すぐにカッとなる	0	1	2	3	4
41	知らない人といると居心地が悪い	0	1	2	3	4
42	イライラする	0	1	2	3	4
43	死にたいと考えることがある	0	1	2	3	4
44	人目を気にしすぎる	0	1	2	3	4
45	適量以上に酒（アルコール）を飲んでしまう	0	1	2	3	4
46	酔っぱらうことが好きである	0	1	2	3	4
47	自制心を失い、暴力を振るうのではないかと心配である	0	1	2	3	4
48	授業へのやる気を維持するのが難しい	0	1	2	3	4
49	飲酒が原因で後悔したことがある	0	1	2	3	4
50	しばしば口論になる	0	1	2	3	4
51	泣くことがよくある	0	1	2	3	4
52	学業についていけない	0	1	2	3	4
53	（暴力などで）他人を傷つけることを考える	0	1	2	3	4
54	食べる量を減らすほど、自己評価があがる	0	1	2	3	4
55	誰も自分のことを理解してくれないと感じる	0	1	2	3	4
