# Supplementary material for: Lingering effects of COVID-19 on the mental health of first-year university students in Japan
Source: PLoS One. 2022 Jan 12;17(1):e0262550. doi: 10.1371/journal.pone.0262550 (PMC8754334; doi:10.1371/journal.pone.0262550)
Supplement: Attachment — Submitted filename: Response to Reviewers.docx [file pone.0262550.s002.docx]

Dear editor:

I would like to resubmit the manuscript titled “Lingering effects of COVID-19 on the mental health of first-year university students in Japan” for publication in PLOS ONE. The manuscript ID is PONE-D-21-31952.

Thank you for your thoughtful comments. I also appreciate the helpfulness of the reviewer’s comments. Per your suggestion, I have integrated the recommended references into the “Introduction.” I will also provide the entire CCAPS-Japanese questionnaire as a supplementary file. Per the reviewer’s comments, I have conducted multiple regression analysis to control for potential confounders, calculated Cronbach’s alpha, and deepened our discussion.

Best regards,

Response to Reviewer 1:

I deeply appreciate your supportive comments. I have edited my previous manuscript, and my responses to your suggestions are as follows. I have also highlighted the corresponding text in the manuscript.

**Introduction**

**Comment:**

**Please explain why it is specifically targeted at first-year college students rather than university students as a whole. Please describe the change in the proportion of anxiety, depression, suicide, violence and other tendencies across the country over the five years before 2019 in order to confirm that the change in numbers was the effect of the COVID-19 pandemic, not the long-standing culture of other societies.**

Response: Per your suggestion, I explained why the study is specifically targeted at first-year university students (P. 6 L. 97–99).

In Japan, there is no national survey on anxiety, depression, and violence. Because I only have access to national surveys on suicide, we described the change in the suicide rates (P. 4 L. 49–53).

**Methods**

**Comment:**

**The authors are recommended to use regression analysis to control confounding factors, for example, gender, department, and family income. Please provide more information about psychometric properties of CCAPS-Japanese.**

Response: Thank you for your kind advice. I have conducted multiple regression analysis using biological sex and department, and the results are shown in Tables 3 and 4 in the “Correlation of demographic characteristics and the eight CCAPS-Japanese subscales” and “Discussion” section (P. 17 L. 305–P. 18 L. 314 and P. 21 L. 374–377). We did not ask the participants about family income.

We have also described more precisely the description of the CCAPS (P. 8 L. 138–141) and submitted the entire CCAPS-Japanese questionnaire as a supplementary file.

**Results**

**Comment:**

**Please present the student's department composition in order to reduce the interference of the department with emotional perception. Line 163 I wonder why the author thought Η2 = .09 was very small. According to your criteria, 0.09 was medium. Please add the percentages after the number of students.**

Response: Thank you for your helpful comments. I have presented the composition of the students by academic department in Table 1.

Per your suggestion, I have modified the description of the effect size evaluation.

Based on your suggestion, I have demonstrated the percentage data as well as numbers in Table 5.

**Discussion**

**Comment:**

**Please discuss the impact of differences in annual response rates on results. In addition, what is the proportion of the whole school to fill in the questionnaire? How is the questionnaire sent to everyone? Was there a selection bias? Line 212-213 I am confused that the worst depression indicating more depressed or less? For students, the pressure of online courses may only appear in a month or two, after which students will find that classes can be mixed up without going out to save a lot of traffic time. Moreover, they might feel that the line class industry more relaxed due to less social stress.**

Response: I appreciate your suggestion. Although there was a difference in the response rate among the three time points, there were no significant differences in the breakdown by sex or academic department in each group, and these percentages were similar to those of Gifu University as a whole. In addition, there was no intention to access the survey. Therefore, I determined that our study did not have any selection bias (P. 16 L. 274–276).

The ratio of first-year students to all students is shown in the “Methods” section (P. 6 L. 115––P. 7 L. 116).

Instructions on how students were asked to submit the questionnaire were included in the “Methods” section (P. 7 L. 116–121).

I apologize for the confusion regarding the wording for depression. I have now clearly stated, “highest scores” for anxiety and depression (P. 16 L. 278).

I edited the discussion regarding online classes as you suggested (P. 17 L. 301–304).
